# Pharmacological analysis of dopamine modulation in the *Drosophila melanogaster* larval heart

**DOI:** 10.1002/phy2.20

**Published:** 2013-06-26

**Authors:** Josh S Titlow, Jenna M Rufer, Kayla E King, Robin L Cooper

**Affiliations:** 1 Department of Biology and Center for Muscle Biology, University of KentuckyLexington, KY, 40506-0225; 2 Department of Biology, Berea CollegeBerea, KY, 40403; 3 Agricultural Biotechnology, University of KentuckyLexington, KY, 40506-0225

**Keywords:** Dopamine, *Drosophila melanogaster*, heart, larva, pharmacology

## Abstract

Dopamine (DA) and other neurotransmitters affect nonneuronal tissues in insects by circulating in the hemolymph. In several organisms, DA has been shown to modulate distinct aspects of cardiac function but the signal transduction pathways that mediate dopaminergic effects on the heart are not well characterized. Here, we used a semiintact *Drosophila melanogaster* larva preparation and drugs targeting DA receptors and canonical second messenger pathways to identify signaling cascades that mediate the effect of DA on a myogenic heart. DA has a positive chronotropic effect that is mimicked by SKF38393 (type-1 DA receptor agonist) and quinpirole (type-2 DA receptor agonist). SCH23390 and spiperone (type-1 and type-2 DA receptor antagonists) are moderately effective at inhibiting DA's effect. An adenylate cyclase inhibitor (SQ,22536) is also effective at blocking the stimulatory effect of DA but the drug has its own dose-dependent effect. Activation of protein kinase C with a diacylglycerol analog has a stimulatory effect on heart rate (HR). These results suggest that (1) both DA receptor subtypes are expressed in third instar larva cardiac myocytes to increase HR in response to rising levels of DA in the hemolymph, and (2) canonical second messenger pathways modulate HR in *D. melanogaster* larvae. Having these disparate signaling cascades converge toward a common modulatory function appears redundant, but in the context of multiple cardioactive chemicals this redundancy is likely to increase the fidelity of signal transduction.

## Introduction

Dopamine (DA) is a well characterized neurotransmitter that also exhibits modulatory effects on peripheral tissues. Cardiac function is influenced by DA in several species, for example, *Periplaneta americana* (Collins and Miller 1977), *Ligia exotica* (Yamagishi et al. 2004b), *Tapes watlingi* (de Rome et al. 1980), *Drosophila melanogaster* (Zornik et al. 1999), *Canus lupus* (Chen et al. 2007), *Cavia porcellus* (Habuchi et al. 1997), *Mus musculus* (Asghar et al. 2011), and *Homo sapiens* (Cosyns et al. 2013). Chronic use of DA prodrugs (e.g., l-dopa) has been linked to cardiac valve dysfunction in man (Delgado et al. 2012). Though DA receptors have been identified in mammalian cardiac tissue (Cavallotti et al. 2010; Tonnarini et al. 2011), pharmacological analysis of the effects of DA on heart rate (HR) and other aspects of cardiac function are lacking. Doing so will increase our understanding of how the cardiac rhythm is modulated and how it is affected by systemic DA homeostasis.

The larval *D. melanogaster* heart is a myogenic tube that spans the rostral:caudal axis of the animal (Gu and Singh 1995). Hemolymph is drawn into the heart through ostia in the posterior pump (which is analogous to a ventricle) and circulated through an aorta back into the visceral lumen. Similarities in the developmental genetics (Bodmer 1995; Bodmer and Venkatesh 1998) and physiology (Choma et al. 2011) between *D. melanogaster* and human hearts make the larval heart an insightful model system.

DA has a positive chronotropic effect (meaning change in HR) on the adult and pupal heart (Johnson et al. 1997; Zornik et al. 1999). To investigate the molecular mechanisms mediating cardiac dopaminergic effects, we used the semi-intact *Drosophila* larva preparation (Cooper et al. 2009). One advantage of this preparation for pharmacological analysis is that the heart is quickly isolated from the nervous system and other sources of modulatory input. Because DA homeostasis is often manipulated systemically to study larval behavior (Neckameyer and Bhatt 2012) we are also interested in the effects of DA on cardiac function.

In the nervous system, and in smooth muscle, dopaminergic modulation proceeds through canonical G protein coupled receptor (GPCR) pathways (Neve et al. 2004). Arthropod DA receptors exhibit strong functional and pharmacological similarities to vertebrate receptors (Mustard et al. 2005; Yuan and Lee 2007). Four DA receptors have been described in *D. melanogaster*. Based on sequence identity and cAMP accumulation assays they can be classified as type-1 (DopR, DopR2, DopEcR) or type-2 (D2R) (Gotzes et al. 1994; Sugamori et al. 1995; Gotzes and Baumann 1996; Han et al. 1996). Type-1 DA and type-2 DA receptors are either positively or negatively coupled to adenylate cyclase through stimulatory and inhibitory G protein alpha subunits. Type-2 DA receptors are also known to function through protein kinase C (PKC) and calcium-dependent pathways (Yan et al. 1999). The degree to which GPCRs activate phospholipase-C and other second messenger cascades in *D. melanogaster* hearts is not completely understood and we are far from understanding how information from multiple signaling pathways is integrated. The aim of this study was to determine if vertebrate drugs targeting DA receptors and second messengers have an effect on this preparation with the long-term goal of dissecting interactions between multiple pathways.

## Materials and Methods

### HR assay

A Canton S. strain that has been isogenic in the lab for several years was used for all experiments. Flies were maintained on a 12 h light:dark cycle in bottles at medium density and fed standard cornmeal fly food (Bloomington stock center recipe). Early third instar larvae were pinned ventral side up on a glass plate and dissected in a droplet of HL3 saline (Stewart et al. 1994): (in mmol/L) 70 NaCl, 5 KCl, 20 MgCl_2_, 10 NaHCO_3_, 1 CaCl_2_, 5 trehalose, 115 sucrose, 25 N,N-Bis-(2-hydroxyethyl)-2-aminoethane sulfonic acid (BES). Note the following modifications: pH was decreased from 7.2 to 7.1 and BES buffer was increased from 5.0 mmol/L to 25.0 mmol/L to maintain stable pH. All recordings were made at room temperature (21–23°C) between 9 and 5 pm.

The larva dissection was first described by Gu and Singh (1995). Early third instars were opened by an incision in the ventral midline and visceral organs were removed without touching the heart. After recovering from surgery for 5 min the heart was visualized through a dissecting microscope and the baseline HR was measured by directly counting contractions in the posterior “heart” region. The saline was then carefully removed and exchanged with the various drug solutions. Counts in the new solution were taken 1 min after the exchange to allow the heart time to adjust after mechanical agitation, and for the ninth minute after applying the solution to determine the duration of modulatory effects. Hearts that did not beat continuously or stopped beating at the end of the experiment were not included in our analyses. As a control for the solution change, HR was measured after exchanging saline with fresh saline.

### Pharmacology

SCH23390, SKF38393, Quinpirole, SQ22536, and phorbol-12-myristate-13-acetate (PMA) were purchased from Sigma Aldrich (St. Louis, MO). Spiperone was purchased from TOCRIS (Minneapolis, MN). DA HCl and each of the saline salts were purchased from Sigma. DA was weighed out and prepared daily. The other drugs were prepared from stock solutions. Lipophilic drugs were dissolved in saline solutions containing less than 1.0% dimethyl sulfoxide (DMSO). Saline containing 1.0% DMSO did not have an effect on larval HR.

### Analysis

Heart rates were determined by counting the number of contractions observed in the posterior region of the heart (between seventh and eight abdominal segments). Contractions were counted by visual inspection through a dissection microscope. The rates measured after drug treatment were normalized to the rate measured before drug treatment (baseline). Normalized values were then pooled for each treatment and the two-tailed Student's *t*-test (Sigma Plot, 12.0) was used to compare drug treatments to saline treatments (control) and to compare different concentrations of drug treatments. Data points depict the mean and SEM for each treatment at a given 1-min interval during the experiment. Sample sizes for each experiment are indicated in the figure legends.

## Results

### DA increases larval HR

**Figure 1:**
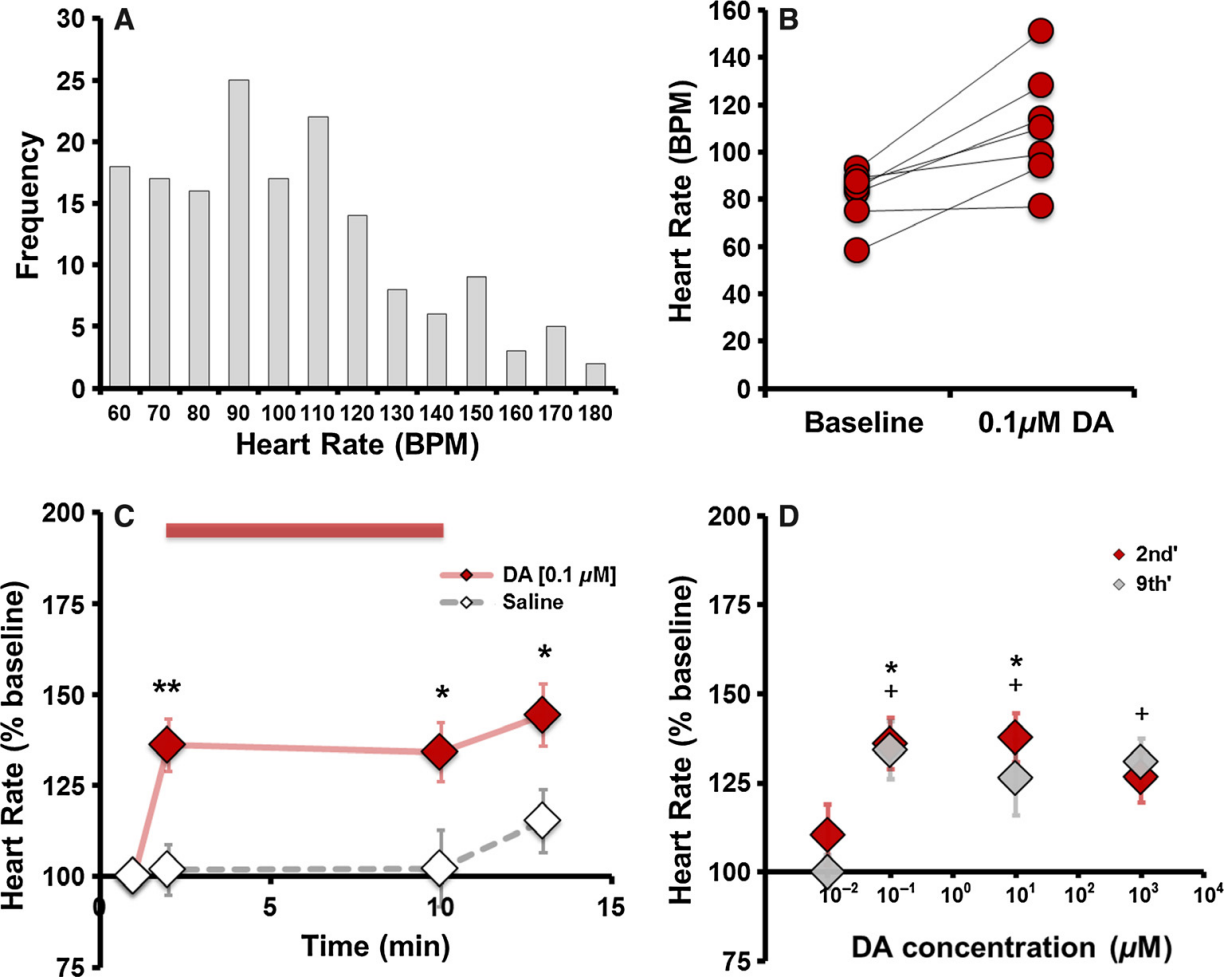
Dopamine (DA) has a positive chronotropic effect on larval heart rate (HR). (A) Baseline HR counts from all experiments, that is, 5 min after dissection and prior to application of drugs. (B) HR counts from seven individual experiments, before and 2 min after the saline was exchanged with 0.1 μmol/L DA. (C) Mean HR (normalized to baseline) plotted with time to show the full time course of the experiments (*n* = 7; **P = 0.005 and *P = 0.03 compared to saline changes, Student's *t*-test). The red bar depicts how long preparations were incubated in the drug solution. (D) Mean HR counts from the second and ninth minute of the experiment in response to different DA concentrations (*n* > 7 for each concentration; *P < 0.05 at 2nd minute, +*P* < 0.05 for the 9th minute compared to saline changes at those times, Student's *t*-test).

The average baseline HR measured in dissected third instar larvae was 98.9 ± 2.5 beats per minute (*N* = 164). The distribution of baseline HRs in this preparation was skewed toward lower frequencies and the range exhibited threefold variation (Fig. 1A). Surgical and environmental differences explain a portion of this variation. Previous reports in nondissected flies show that up to 25% of the variation can be attributed to genotypic differences (Robbins et al. 1999). Statistical analyses were performed on baseline-normalized values to account for this variation.

On average 0.1 μmol/L DA increased HRs 36.02% ± 7.15% above baseline (Fig. 1). At this concentration HR increased in each individual experiment (Fig. 1B). DA caused a rapid rise in HR upon exposure and maintained a heightened level during the 0.1 μmol/L treatment (Fig. 1C). In most cases the increase persisted for at least 10 min and was not immediately washed out by saline after the treatment. The dose–response results (Fig. 1D) would suggest that 0.1 μmol/L DA reaches a saturation effect in increasing HR as higher concentrations did not produce significantly higher rates.

### Type 1 and 2 DA receptors mediate dopaminergic modulation of larva HR

**Figure 2:**
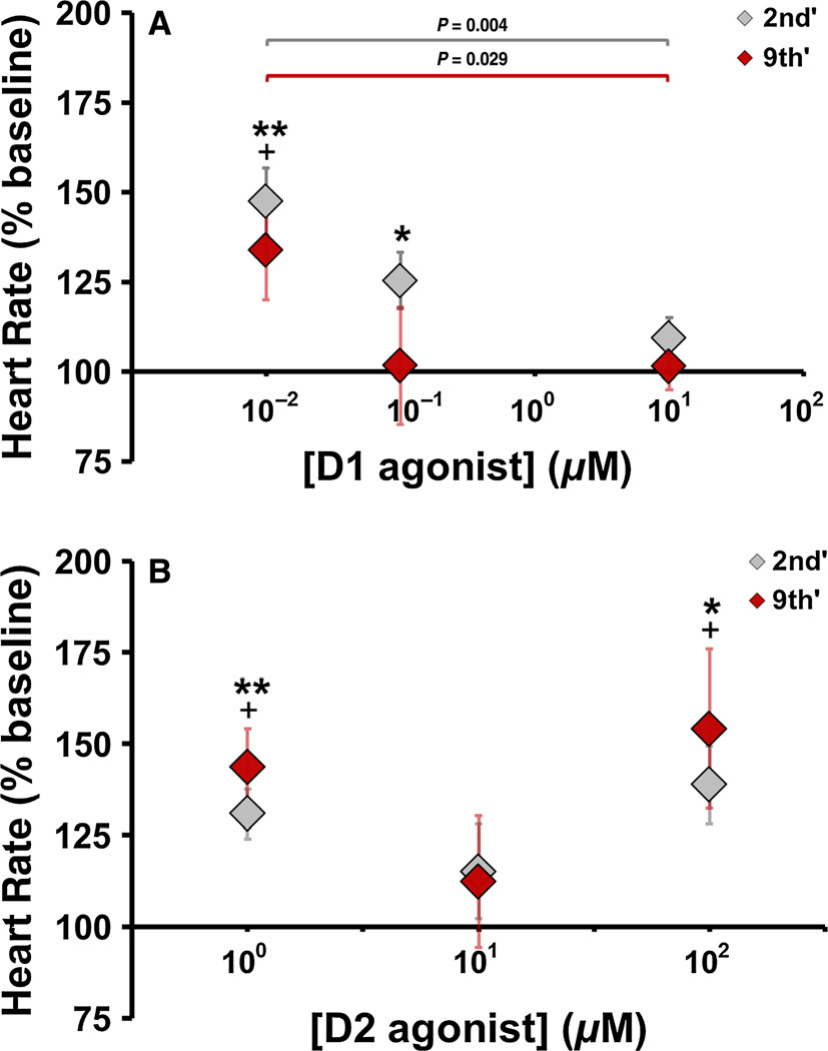
Synthetic vertebrate agonists for the two dopamine (DA) receptors increase HR in 3rd instar larvae. Agonist application regimens and HR counts were identical to DA experiments in Figure 1C, that is, change is shown relative to baseline 2 and 9 min after the drugs were applied. (A) SKF38393 (type-1 DA receptor agonist) and (B) quinpirole (type-2 DA receptor agonist) have positive chronotropic effects (**P < 0.005, *P < 0.05 compared to 2nd minute of saline treatment, Student's *t*-test, +*P* < 0.05 compared to 9th minute of saline treatment, Student's *t*-test; *N* > 5 different individuals for each treatment). The magnitude of the effects is statistically similar to DA but there are subtle differences in temporal and dose response. Efficacy of the type-1 DA receptor agonist decreased at higher concentrations.

Pharmacological approaches were used to investigate the mechanisms of dopaminergic modulation in this system. Synthetic vertebrate DA receptor agonists and antagonists are known to bind to *Drosophila* DA receptors and have pharmacological effects comparable to vertebrates in heterologous expression systems (Gotzes et al. 1994). In *Drosophila* cell culture (Yuan and Lee 2007) and in the intact nervous system (Yellman et al. 1997) several common vertebrate DA receptor drugs have been used to correlate specific DA receptors with a modulatory effect. The type-1 and type-2 DA receptor agonists used in these experiments were SKF38393 and quinpirole. SKF38393 was applied to larval hearts at 0.01, 0.1, and 10.0 μmol/L concentrations. At each concentration there was an initial dose-dependent increase (20–70%) in HR followed by a return to rates that were 1–36% above baseline (Fig. 2A).

The type-2 agonist quinpirole caused an initial dose-dependent increase in HR that grew during the incubation to 12–53% above baseline (Fig. 2B). This was in contrast to the chronotropic effect of SKF38393, which diminished during the course of treatment (*P* = 0.03 at 10 μmol/L). Also the acute chronotropic effect of SKF38393 was smaller at higher concentrations. These results suggest that the type-1 DA receptors desensitize in response to prolonged exposure to ligand. Mechanisms of DA receptor desensitization have been described in neuronal tissues (Rex et al. 2008; Beaulieu and Gainetdinov 2011) but further pharmacological characterization is needed to confirm this phenomenon in *D. melanogaster* cardiac cells.

**Figure 3:**
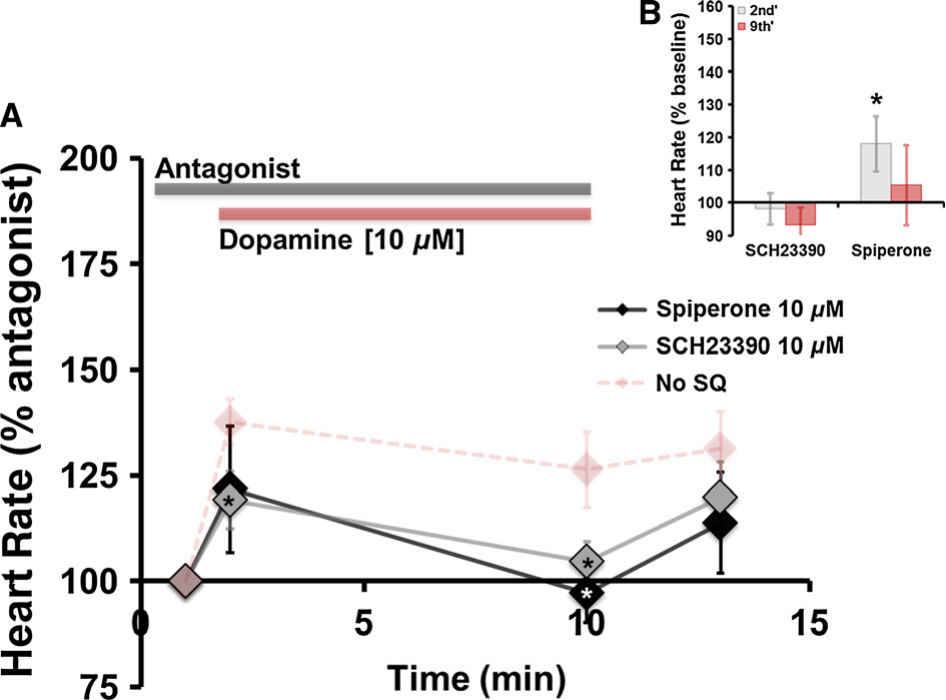
Type-1 and type-2 DAR antagonists partially block the modulatory effect of dopamine (DA) on larval HR. Dissected larva hearts were pretreated with a DA receptor antagonist for 10 min prior to DA application. The dopaminergic increase in HR was partially blocked by either drug 2 min after DA application, and almost completely blocked 9 min after DA application (*P < 0.05 compared to the effect of DA without the antagonist at that time point, Student's *t*-test, *n* > 8 different individuals for each treatment). SCH23390 is a type-1 DA receptor antagonist (gray data points) and spiperone is a type-2 DA receptor antagonist (black data points). The dopaminergic effect without pretreatment with antagonists is shown in light red. (B) SCH23390 alone did not have an effect on HR at either time point. Spiperone caused a slight but significant increase in HR that diminished before DA was added.

Antagonists for both DA receptor subtypes were moderately effective at blocking the effect of DA. For those experiments the dissected preparation was pretreated with either antagonist before adding a solution containing DA and the antagonist. HRs for the DA treatment were normalized to values recorded at the end of the pretreatment. The effect of DA (10 μmol/L) on HR was completely inhibited by antagonists for either DA receptor 9 min after DA was applied (Fig. 3A). The type-1 DA receptor antagonist (SCH23390, 10 μmol/L) significantly blocked the immediate effect of DA but the type-2 antagonist (spiperone, 10 μmol/L) was not as effective at this time point (i.e., 2 min after DA was applied). Spiperone initially increased HR during pretreatment but the rate returned to baseline levels before DA was added (Fig. 3B). Possible explanations for this result are that the drug has off-target effects or that the drug inhibited the function of a constitutively active DA receptor. Constitutively active DA receptors have been identified in *Aplysia* heart (Barbas et al. 2006) and in mammalian nervous system (Tiberi and Caron 1994; Charpentier et al. 1996).

### Adenylate cyclase and PKC are involved in modulation of HR

**Figure 4:**
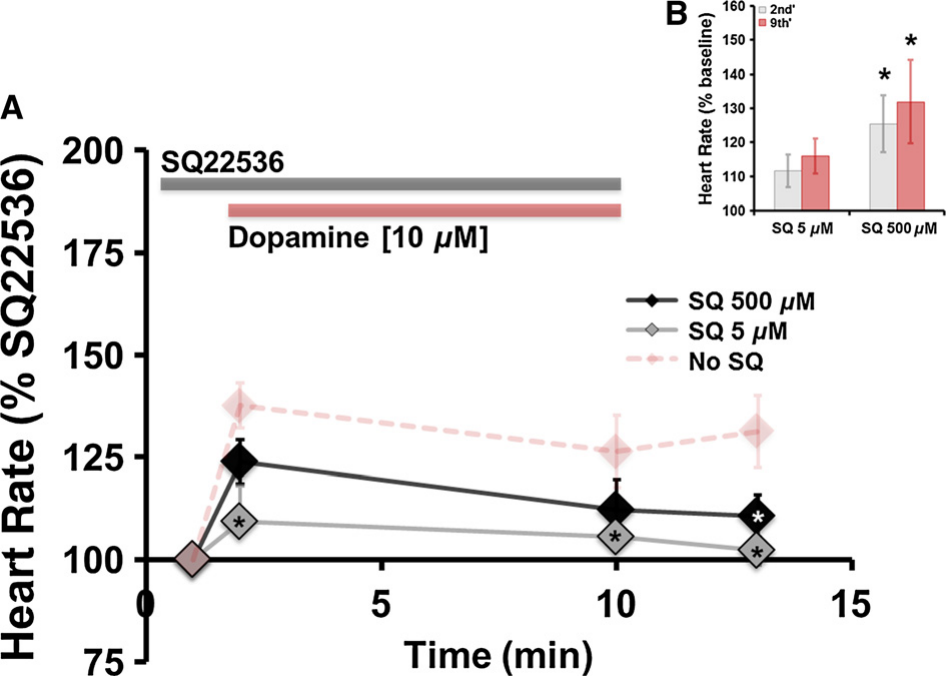
Dopaminergic modulation of larval HR is mediated by adenylate cyclase. Dissected hearts were pretreated for 10 min with SQ22536 (adenylate cyclase inhibitor) as in Figure 3. HRs shown here were normalized to the rate recorded at the end of SQ22536 treatment. (A) At 500 μmol/L (black data points) and at 5 μmol/L (gray data points) the drug attenuated the effect of dopamine (DA). Inhibition was only statistically significant for the lower concentration (*P < 0.05 compared to DA alone (shown in light red), Student's *t*-test: *n* > 9 individuals for each concentration). (B) At both concentrations SQ22536 alone increased HR relative to saline treatment (*P < 0.05, Student's *t*-test, pretreatment rates from preparations in [A]).

To determine if DA acts on the heart through classical stimulatory GPCR pathways, the vertebrate adenylate cyclase inhibitor SQ22536 (SQ) was tested in the same manner as the DA receptor antagonists, that is, the drug was applied for 10 min before applying it in solution with 10 μmol/L DA. HRs were measured at the end of this treatment and later time points were normalized to those pretreatment rates. Under these conditions the modulatory effect of DA (10 μmol/L) was significantly inhibited by 5 μmol/L SQ (Fig. 4A). Oddly SQ was less effective at 500 μmol/L and at both concentrations SQ alone had a stimulatory effect on HR (Fig. 4B). Though the drug clearly inhibits dopaminergic modulation in this context, we are unable to rule out the possibility of off-target mechanisms.

**Figure 5:**
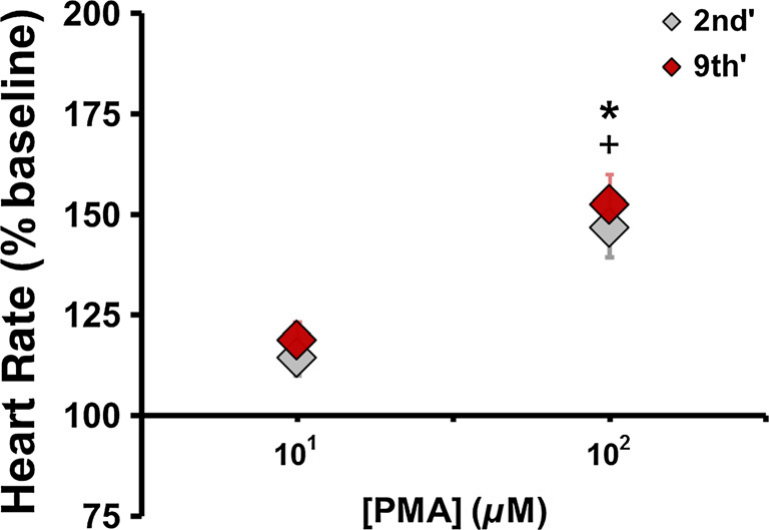
Activation of protein kinase C (PKC) has a positive chronotropic effect on HR. Phorbol-myristate-acetate (PMA), a cell permeable diacylglycerol analog, was used to determine if PKC acts as a second messenger in modulation of insect HR. The stimulatory effect of PMA was much stronger at the higher concentration (*P < 0.005 after 2nd minute compared to 10 μmol/L, +*P* < 0.005 after the 9th minute compared to 10 μmol/L; *n* > 10 different animals for both concentrations).

The diacylglycerol (DAG) analog PMA was used to determine if PKC is involved in modulation of larval HR. This drug consistently increased HR and was more effective at 100 μmol/L than at 10 μmol/L (Fig. 5). At both concentrations the effect lasted for 10 min and was not immediately washed out.

### Calcium ion reduction inhibits dopaminergic modulation of larval HR

**Figure 6:**
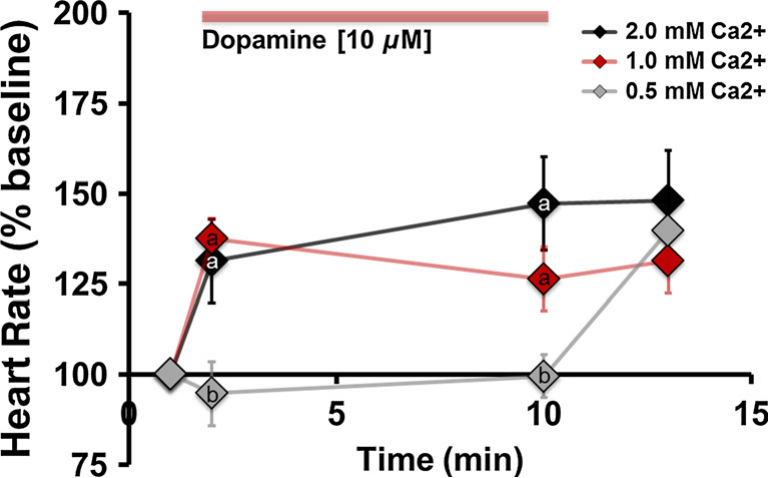
The modulatory effect of dopamine (DA) on heart rate (HR) is correlated with extracellular Ca^2+^ levels. 1.0 mmol/L (red data points) is the normal [Ca^2+^] used for HL3 (hemolymph-like) fly saline. In low [Ca^2+^] conditions (gray data points) the positive chronotropic effect of DA was not observed. In high calcium (black data points) the effect of DA is unaffected, though the persistence of the dopaminergic effect is slightly stronger in this condition. Data points with different letters exhibited significantly different rates (*P* < 0.05, Student's *t*-test; *n* > 6 for each [Ca^2+^] tested). The solution used for dissection contained the same [Ca^2+^] as the treatment solution.

To test the hypothesis that Ca^2+^ influx is a factor in dopaminergic modulation of larval HR, we experimented with various levels of [Ca^2+^]_o_. The normal HL3 saline contains 1.0 mm CaCl_2_ (Stewart et al. 1994). At 0.1 mm Ca^2+^ the hearts did not beat, but changing the saline to 0.5 mm Ca^2+^ revived them from cardiac arrest (*n* = 5). In this low calcium solution DA (10 μmol/L) did not have an effect on HR (Fig. 6). High calcium saline (2.0 mm) did not change the effect of DA at any point. Therefore, calcium influx is necessary for dopaminergic modulation of HR and it appears that [Ca^2+^]_o_ contributes its maximum input at 1 mmol/L. Higher [Ca^2+^]_o_ is known to substantially increase HR and further modulatory effects are difficult to ascertain at higher frequencies (Desai-Shah et al. 2010). After washing away DA in the low calcium solution there was a 40% increase in HR. Slight increases after washout were observed in other treatments (Figs. 1C and 3) but this phenomenon was accentuated in this condition. One explanation is that after several minutes without calcium the tissue developed an increased sensitivity to mechanical stress, causing an elevated response to the solution change.

## Discussion

### Positive chronotropic effect of DA on *D. melanogaster* larval hearts

Endogenous DA levels fluctuate in response to environmental cues and an animal's state of arousal (Noguchi et al. 1995). In insects DA modulates peripheral organs by circulating in hemolymph at concentrations in the micromolar range (Matsumoto et al. 2003). The source of hemolymph DA is debatable but it likely originates from hypodermal cells that secrete DA to harden the cuticle (Wright 1987; Friggi-Grelin et al. 2003) or from neurohemal axon terminals (Buma 1988) and varicose projections within the nervous system (Helle et al. 1995), as is the case in other invertebrates. Regardless of the source, our data show how a sudden increase in DA has a positive chronotropic effect on the semi-intact larval heart in *D. melanogaster*. This effect has not been described in larvae but Zornik et al. (1999) reported a positive chronotropic effect in adults and a negative effect in pupae. Two factors that may have led to different findings between the two studies were the developmental stage and genotype. Here, early third instar larvae from the Canton S line were used whereas the previous study used Oregon R flies in the “wandering” third instar stage. Using intact P1 pupal stage from the Canton S line, Johnson et al. (1997) observed that DA has a positive chronotropic effect. Similar developmentally specific dopaminergic effects on HR have also been reported in the sea roach, *L. exotica* (Yamagishi et al. 2004a). Moreover DA has a positive chronotropic effect on the cockroach heart (Collins and Miller 1977). The data suggest that DA is regulating HR by modulating pacemaker activity in cardiac myocytes (Johnson et al. 2002), but whether modulation occurs directly in the myocardial cell layer or indirectly through the epicardium is unclear (Su et al. 1999). We did not measure contractile force generated by the cardiac tube but experiments in *Limulus polyphemous* have shown that DA can have a positive ionotropic effect (change in contractile function) on invertebrate heart muscle and that the effect is mediated by cyclic-AMP and PKC (Groome and Watson 1989).

### Canonical second messenger pathways involved in fruit fly HR modulation

Using SQ and ion substitution we showed that DA exerts its positive chronotropic effect on HR through adenylate cyclase and calcium influx (Figs. 4 and 6). Coupling of a *D. melanogaster* type-1 DA receptor to increases in intracellular Ca^2+^ and cyclic-AMP through G proteins has been demonstrated in *Xenopus* oocytes (Reale et al. 1997). The effect of cyclic-AMP is subtle and confounded by the fact that SQ increased HR. In pupal hearts a cyclic-AMP analog (8-bromo-cAMP) had a very small stimulatory effect (9.5%) and forskolin did not significantly affect HR (Johnson et al. 2002). Mutations in adenylate cyclase (*rutabaga*) and cAMP phosphodiesterase (*dunce*) did not significantly alter the stimulatory effects of cardioactive molecules (Johnson et al. 2002). However, there is strong genetic evidence that indicates the involvement of calcium and phospholipase C in modulation of HR (Johnson et al. 2002) and our pharmacological data are consistent with those findings.

We showed that direct activation of PKC has a dose-dependent stimulatory effect on larval HR (Fig. 5). The PKC pathway could modulate HR by targeting calcium channels. In rat ventricular myocytes an l-type calcium current is modulated through a PKC-dependent pathway (Chen et al. 2012). Calcium handling in *Drosophila* myocytes exhibits many of the same physiological properties observed in mammalian myocytes. l-type Ca^2+^ channels enable periodic waves of calcium influx (Gu and Singh 1995). Intracellular calcium is in turn buffered by sarcoplasmic/endoplasmic reticulum Ca^2^^+^ -ATPase (Sanyal et al. 2006) and a sodium/calcium exchanger (Desai-Shah et al. 2010). Larval HR is positively correlated with extracellular calcium, for example, decreasing [Ca^2+^]_o_ from 1.0 mmol/L to 0.5 mM decreases HR by over 50%, and increasing [Ca^2+^]_o_ from 1.0 to 2.0 mmol/L increases HR by 40% (Desai-Shah et al. 2010). Although the experiments reported here did not directly address activation of PKC signaling by DA, evidence from rat myocytes (Li et al. 2009) and *Aplysia* sensory neurons (Dunn et al. 2012) indicate that DA modulates the function of those cells through a PKC-dependent pathway.

Though DA and the DA receptor agonists used here were effective at doses that are likely below the threshold to exert off-target effects, our experiments do not completely rule out the possibility that DA was acting through an adrenergic or other aminergic receptor. Norepinephrine and an alpha-adrenergic receptor agonist have been shown to increase pupal HR in *D. melanogaster* (Johnson et al. 2002). However, there are no true adrenergic receptors in *D. melanogaster (*Evans and Maqueira 2005). These molecules are believed to act through octopamine or tyramine receptors, which exhibit pharmacological properties similar to adrenergic receptors (Bayliss et al. 2013).

### Future studies and impact

In *D. melanogaster* a leak current from an outward rectifying potassium channel (ORK1) regulates HR by controlling membrane excitability and in turn the slow diastolic depolarization phase (Lalevee et al. 2006). It is possible that DA influences HR through protein kinases that inactivate ORK1 through phosphorylation. Indeed it was shown that HR increased when expression of this channel was knocked down (Lalevee et al. 2006). The current work establishes a system for addressing hypotheses about the mechanisms of aminergic modulation of the heart using electrophysiological techniques and transgenic flies.

In mammals the effects of DA on cardiovascular function have been studied extensively. Chronotropic, ionotropic, and pressor effects have been demonstrated in guinea pigs, rabbits, dogs, and in humans (Tsai et al. 1967; Wakita 2007). In several instances the results are contradictory and complicated by the fact that DA acts on smooth muscle as a vasodilator, it modulates parasympathetic innervations to the heart, and can be taken up by neurons and converted to norepinephrine. Nonetheless it has been shown that each DA receptor subtype is expressed in mammalian hearts (Cavallotti et al. 2010; Tonnarini et al. 2011), giving some support to the idea that DA or DA receptor agonists could modulate the mammalian heart directly. A current concern is that extended use of DA prodrugs (e.g., l-dopa) and DA receptor agonists for neurobiological disorders has been associated with cardiac valve dysfunction (Delgado et al. 2012; Trifiro et al. 2012). Though circulating DA levels are typically not high enough to activate DA receptors (Zeng and Jose 2011), these pharmacological agents seem to influence cardiac function through DA pathways. The larval heart has a pair of intracardiac cells that function as a valve (Zeitouni et al. 2007; Lehmacher et al. 2012), so this system could potentially be used to address the molecular mechanisms that cause this valvular dysfunction associated with DA treatments.

### Conclusion

Our pharmacological analysis indicates that the chronotropic effect of DA is mediated by functionally conserved G protein coupled DA receptors and canonical second messenger pathways. The results also indicate that calcium flux is an important element of dopaminergic modulation in the heart. These data can guide future studies that address interactions between signaling pathways and homeostatic changes to monoamine signaling using the genetic tools available in this system.
